# User Experience and Potential Health Effects of a Conversational Agent-Based Electronic Health Intervention: Protocol for an Observational Cohort Study

**DOI:** 10.2196/16641

**Published:** 2020-04-03

**Authors:** Marian Z M Hurmuz, Stephanie M Jansen-Kosterink, Harm op den Akker, Hermie J Hermens

**Affiliations:** 1 eHealth Group Roessingh Research and Development Enschede Netherlands; 2 Biomedical Signals and Systems Group Faculty of Electrical Engineering, Mathematics and Computer Science University of Twente Enschede Netherlands

**Keywords:** virtual coaching, effectiveness, user experience, evaluation protocol, older adults, adults, type 2 diabetes mellitus, chronic pain, healthy lifestyle

## Abstract

**Background:**

While the average human life expectancy has increased remarkably, the length of life with chronic conditions has also increased. To limit the occurrence of chronic conditions and comorbidities, it is important to adopt a healthy lifestyle. Within the European project “Council of Coaches,” a personalized coaching platform was developed that supports developing and maintaining a healthy lifestyle.

**Objective:**

The primary aim of this study is to assess the user experience with and the use and potential health effects of a fully working Council of Coaches system implemented in a real-world setting among the target population, specifically older adults or adults with type 2 diabetes mellitus or chronic pain.

**Methods:**

An observational cohort study with a pretest-posttest design will be conducted. The study population will be a dynamic cohort consisting of older adults, aged ≥55 years, as well as adults aged ≥18 years with type 2 diabetes mellitus or chronic pain. Each participant will interact in a fully automated manner with Council of Coaches for 5 to 9 weeks. The primary outcomes are user experience, use of the program, and potential effects (health-related factors). Secondary outcomes include demographics, applicability of the virtual coaches, and user interaction with the virtual coaches.

**Results:**

Recruitment started in December 2019 and is conducted through mass mailing, snowball sampling, and advertisements in newspapers and social media. This study is expected to conclude in August 2020.

**Conclusions:**

The results of this study will either confirm or reject the hypothesis that a group of virtual embodied conversational coaches can keep users engaged over several weeks of interaction and contribute to positive health outcomes.

**Trial Registration:**

The Netherlands Trial Register: NL7911; https://www.trialregister.nl/trial/7911

**International Registered Report Identifier (IRRID):**

PRR1-10.2196/16641

## Introduction

As a result of socioeconomic development and progression in medicine and education, the average human life expectancy has increased significantly [1,2]. However, the aging population has also led to more older adults living with chronic diseases [2,3]. Although these diseases cannot be cured, their burden on patients can be reduced by adopting a healthy lifestyle [2,4,5]. To enable adoption of a healthy lifestyle, a deep understanding of personal motivation and the person’s economic and social pressures is needed [6,7]. Based on these insights, personalized virtual coaching systems have been developed to support lifestyle changes [8]. For these systems, using multiple coaches is more effective than using a single coach because of the potential positive impact of vicarious persuasion as compared with direct persuasion (persuasion of the crowd instead of directly persuading the person) [9]. This insight has led to the introduction of the Council of Coaches (COUCH), a new concept for virtual coaching [10].

COUCH comprises a council of 5-6 virtual coaches. These coaches inform and motivate the user and discuss different topics about healthy living [10]. COUCH was developed in collaboration with end users, and the feasibility and usability of some parts of COUCH have already been tested in a lab setting (formative evaluations). The next step is to gain, through a summative evaluation, knowledge on the possible working mechanism and potential added value of this coaching system in a real-world setting among the target population [11]. As we do not want to interfere with the ongoing development of COUCH, we decided to develop a mature and simplified version of COUCH ready for testing in a real-world setting. This paper outlines the study protocol for this first test in the real world, which aims to evaluate the user experience with and the use and potential health effects of a fully working COUCH system implemented in a real-world setting among the target population.

## Methods

### Trial Design

This study protocol strictly follows the CONSORT-eHEALTH checklist [12] for the introduction and methods sections. This study is an observational cohort study with a pretest-posttest design. It is explorative and evaluative. The participants will be included for at least 5 weeks and up to a maximum of 9 weeks. The first week will consist of the preparation phase. In this phase, baseline measurements will be collected (T0). The following 4 weeks will consist of the implementation phase (T1). The participants will interact with COUCH during this phase. The last 4 weeks will consist of the facultative follow-up phase (T2). Participants can choose whether they want to interact with COUCH for these additional 4 weeks.

This study will be conducted in 2 countries (the Netherlands and Scotland) and consist of 2 rounds. Each round will include 25 participants per country. During the development phase, the technology and content were tested extensively. Therefore, during this study, we do not expect technical problems. However, if participants encounter minor technical problems during the first round, these problems will be fixed. During both rounds, content will be added to various coaches.

To properly evaluate the effectiveness of technology-supported health services, such as COUCH, in the real world is challenging [13-15], and it is currently increasingly acknowledged among experts that there is an urgent need for more pragmatic study designs to adequately evaluate technology-supported health services [13-16]. Microrandomization could be an appropriate alternative study design. The microrandomized trial was introduced by Klasnja et al [17] to overcome the limitations of current experimental methods, for instance randomized controlled trials, and to supplement the use of behavioral theory to guide the development of just-in-time adaptive interventions. As we are also interested in the effectiveness of the interaction between the user and the virtual coaches, we want to assess the applicability of the virtual coaches and the users’ duration of interaction with the virtual coaches of a fully working COUCH system implemented in a real-world setting among the target population. To assess the users’ interaction with the virtual coaches of COUCH, the interaction with one of the primary coaches (physical activity coach) will be microrandomized: Every time the user starts a conversation with this coach, the initiative of starting the conversation will be based on microrandomization. This microrandomization consists of the following two conditions: (1) The user takes the initiative and chooses the topic of the conversation, or (2) the system takes the initiative and automatically suggests the topic of conversation.

The predefined topics include gathering information about the user, goal setting, strategy selection, learning skills, and feedback and support.

### Participants

The study population will consist of older adults and adults with type 2 diabetes mellitus or chronic pain. For this study, the term older adult is defined as ≥55 years of age, and adult is defined as ≥18 years of age. A potential participant who meets any of the following criteria will be excluded from participating in this study: not able to read and speak Dutch or English, not having a Wi-Fi connection at home, not able to provide informed consent, or not able to see the smartphone or tablet screen clearly.

**Figure 1:**
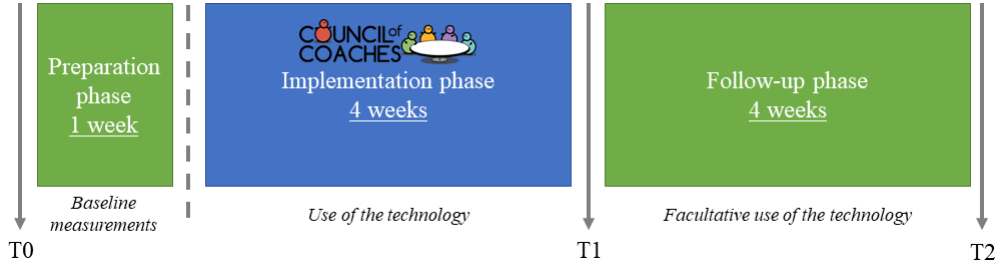
Study procedures for the first and second rounds of this 9-week observational study with a pretest-posttest design.

Eligible older adults will be recruited for the first round from December 2019 through January 2020. Participants will be recruited for the second round from March through April 2020. The first round will start in February 2020. The preparation phase (1 week) will start with an initial visit to the participant’s home or an intake at the researcher’s lab. During this phase, the participants do not interact with COUCH yet, but they will wear sensors for the baseline measurements, and they can keep track of their eating patterns using a food diary. The needed equipment (eg, tablet, smartphone, sensors) will be provided to the participants during this first visit, and they will receive an explanation about the operation of any equipment. Finally, participants will complete the T0 questionnaire. After this first week, the implementation phase will start. The participants will start using the COUCH system for 4 weeks. Thereafter, the second visit will take place at home or at the research location. During this visit, an exit interview will be conducted. The participants will complete the T1 questionnaire, and they will choose whether they want to continue using COUCH for another 4 weeks (the facultative follow-up phase). If they do not want to keep using COUCH, they will return the borrowed equipment to the research staff. After the follow-up phase, all participants will complete the T2 questionnaire. The questionnaires (online or on paper) will be filled in at T0, T1, and T2 (see Figure 1).

### Intervention

**Figure 2:**
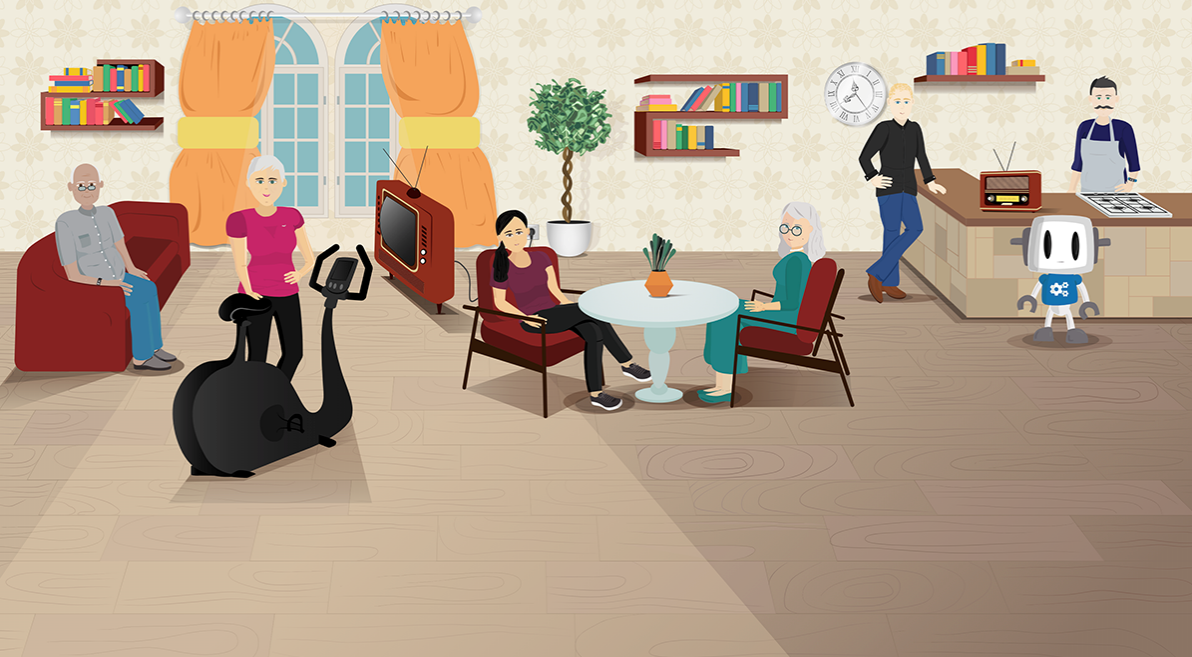
Screenshot of the current test version of the Council of Coaches web application with the chronic pain coach, without a dialogue box (https://www.council-of-coaches.eu/beta/).

**Figure 3:**
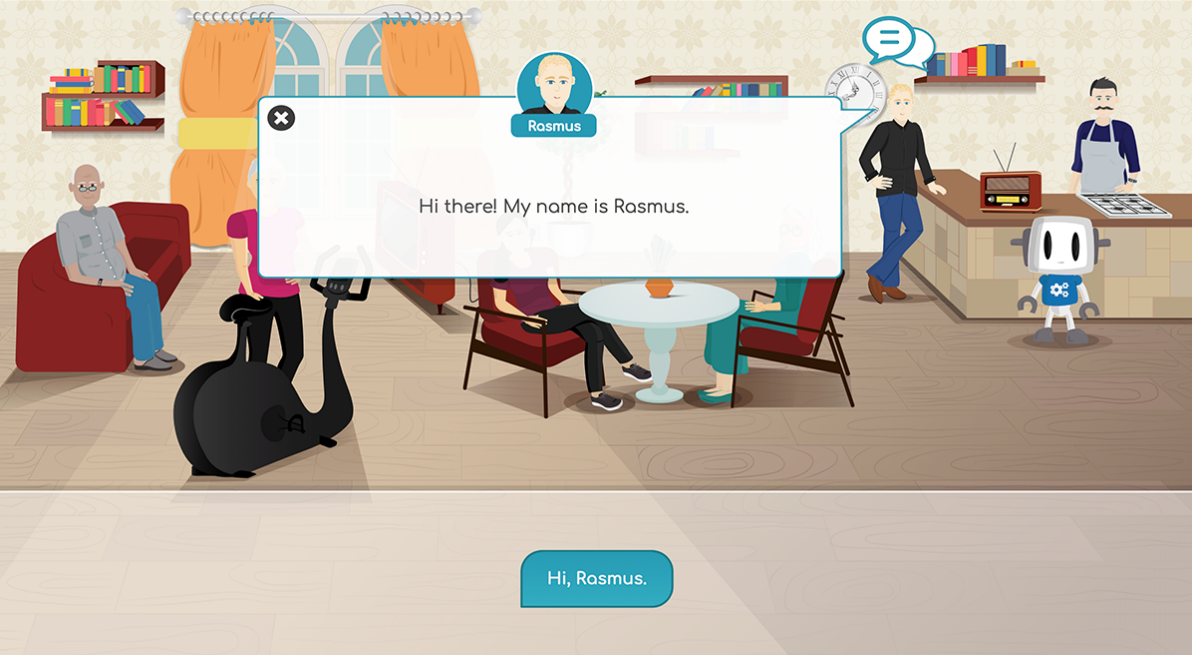
Screenshot of the current test version of the Council of Coaches web application with the chronic pain coach, with a dialogue box (https://www.council-of-coaches.eu/beta/).

The application is a web application, designed and built to run on tablets or computers. This technology is currently under development within the COUCH project (European Union’s Horizon 2020 research and innovation program under grant agreement No. 769553). The application’s main functionality is to provide a friendly and easy-to-use interface that allows users to have natural language dialogues with a group of (5-6) virtual coaches (see Figures 2 and 3). The final COUCH demonstrator will support the following virtual coaches: physical activity, nutrition, social, cognition, peer/support, chronic pain, and diabetes. Depending on the user’s needs and interests, a subset of these coaches can be selected by the user (eg, in the absence of the specific conditions, the chronic pain and diabetes coaches will not be presented to the user).

The content provided by the virtual coaches focuses on physical fitness and nutrition to improve the users’ wellbeing, and the content is based on (Dutch) health guidelines. Both the physical activity and nutrition coaches, which are the primary coaches, can assist the user in their domain in the following ways: providing information on health benefits, setting personalized goals, providing feedback and advice, reflecting on different coaching styles, and assisting with relevant sensor technology.

The secondary coaches (social, cognition, peer/support, chronic pain, and diabetes) interact with the user by providing their points of view on the main topics of activity and nutrition. For example, the social coach may suggest doing group activities outside the house when the user is discussing physical activity with his physical activity coach, while the cognitive coach can provide a memory game to do while grocery shopping for a recipe that the nutrition coach recommended. The peer/support coach is included to be “on the side of the user” and provides encouragement for the user to achieve his/her goals. The secondary coaches, except for the chronic pain and diabetes coaches, can be removed from the council by the user. The interaction with the physical activity coach will be microrandomized [17]. Every time the user starts a conversation with a primary coach, the initiative of the conversation will be taken by the system or given to the user.

The application optionally supports the use of sensor technology, in order to allow personalized feedback and coaching to the users. The physical activity coach will suggest the user wears a Fitbit watch, which is provided by the researchers to all participants, so that she may provide feedback on the user’s actual activity. Similarly, the nutrition coach will ask the user to track dietary consumption through a provided smartphone app and ask the user to enter their weight information either manually or through a connected (smart) scale. Users can talk with their virtual coaches about the use of these devices, and the coaches will explain which data is collected and for what purpose and offer the ability to stop tracking data when the user feels uncomfortable about this.

All of the interactions take place in the comfort of the coaches’ living room (see Figure 2) that includes elements like a radio (playing the coaches’ favorite classical songs), recipe books (that Francois, the nutrition coach, can guide the user through), and a television on which to watch physical exercise examples.

During the first visit (T0), the participants will be trained by the researcher to learn how to interact with COUCH on their tablet, and they will receive a paper manual about COUCH. During the entire evaluation period, there will be a helpdesk available for the participants on working days from 9 am to 5 pm, and the participants will receive a nonpersonalized informative newsletter three times by email to inform them about the project and running evaluation.

### Outcomes

In this study, we will focus mainly on user experience, potential effects on health-related factors, and the use of COUCH during the implementation and follow-up phases. Furthermore, we will examine the demographics, applicability of the virtual coaches, and user’s interaction with the virtual coaches. Table 1 gives an overview of all the questionnaires that will be used during this study. All survey questions in the 3 questionnaires are listed in Multimedia Appendix 1.

**Table 1:** Overview of the questionnaires and when they will be used.

		T0^a^	T1^b^	T2^c^
**User experience**				
	Technology Acceptance Model	–^d^	X	–
	System Usability Scale	–	X	–
	Willingness to pay	–	X	–
**Potential health effects**				
	EQ-5D-5L^e^	X	X	X
	Positive health dimensions	X	X	X
	Self-Management Ability Scale – short version	X	X	X
Demographics		X	–	–
**Applicability of the virtual coaches**				
	Rating scale	X	X	–
	Working Alliance Inventory	–	X	–

^a^Baseline.

^b^After the 4-week implementation phase.

^c^After the 4-week facultative follow-up phase.

^d^Not applicable.

^e^EQ-5D-5L: 5-level EQ-5D questionnaire.

#### User Experience

To determine the user experience, the Technology Acceptance Model [18,19] and System Usability Scale (SUS) [20] will be used. Furthermore, an exit interview will be conducted, and the willingness to pay will be measured. In this study, user experience domains will be used as external variables. In the literature, 4 constructs are found for the user experience of electronic health (eHealth) services. The first is enjoyment. van der Heijden [21] defined perceived enjoyment of a technology as the extent to which fun can be derived from using the system as such. He used 4 questions on a 7-point semantic differentials scale to measure the following 4 items: enjoyable – disgusting, exciting – dull, pleasant – unpleasant, and interesting – boring. The second construct is aesthetics. Lavie and Tractinsky [22] developed and validated a questionnaire to measure perceived website aesthetics. In this study, only classical aesthetics will be used. The third construct is control. In their study, van Velsen et al [23] used 3 control questions from Liu [24] that measure how users perceive the controllability of websites. The fourth construct is trust in technology. This domain is also a predictor for someone’s intention to use technology [23]. van Velsen et al [23] used 4 statements about trust in technology based on the study of Harrison McKnight et al [25] about the impact of consumer trust on intentions to transact with a website.

Perceived usefulness, perceived ease of use, and intention to use will also be used as constructs in this study’s questionnaire. The attitude toward the technology domain will be used as a demographic variable for the secondary outcomes. Both the perceived usefulness and perceived ease of use constructs are derived from Davis [18]. In his study, a new measurement scale for perceived usefulness and perceived ease of use was developed and validated. Both constructs are important when determining the intention to use: the less effort involved in a technology, the more it will be used, and the greater someone’s belief that using the technology would enhance his/her performance, the more it will be used [18,26]. Regarding the intention to use construct, van Velsen et al [23] based this construct on those of Davis et al [19] and Gefen et al [27] and expanded it with one item of their own. Based on the study by van Velsen et al [23], 3 statements were used in this study. Those 3 items were deemed the best to assess the intention to use.

The aesthetics, control, trust in technology, perceived usefulness, perceived ease of use, and intention to use constructs all use statements rated using a 7-point Likert scale, ranging from total disagreement to total agreement.

The SUS will be used to measure the usability of COUCH. Broekhuis et al [28] showed that the SUS is insufficient as a standalone tool for assessing the usability of eHealth technologies. However, another eHealth usability tool is not yet available [28]. The SUS consists of 10 statements with 5 response options that are rated using a 5-point Likert scale ranging from strongly disagree to strongly agree. The SUS score ranges from 0 (worst imaginable) to 100 (best imaginable) points [20].

Qualitative feedback from the participants will be obtained through a short semistructured exit interview at T1 (after interacting with COUCH for 4 weeks). During this interview, participants will be asked to share their ideas about COUCH. We will discuss the advantages, points for improvement, and problems experienced.

Willingness to pay will be measured by asking whether the participants are willing to pay for COUCH, and, if so, how many Euros they are willing to pay.

#### Potential Effect on Health-related Factors

Health effects will be measured through differences in scores within the EQ-5D-5L questionnaire, 6 domains of Positive Health, and Self-Management Ability Scale – short version (SMAS-S). The EQ-5D-5L questionnaire measures quality of life and consists of a descriptive system that includes 5 dimensions (mobility, self-care, usual activities, pain/discomfort, anxiety/depression) and a visual analogue scale. Each dimension has 5 levels, ranging from no problems to extreme problems. With the visual analogue scale, the participants rate their health on a vertical scale, labelled from the worst health you can imagine (0) to the best health you can imagine (100) [29].

Huber et al [30] studied how people think about health. They concluded that the concept of health no longer fits within the definition of the World Health Organization (health as complete wellbeing and absence of disease). The Institute for Positive Health created a tool to gain insight into the positive health of a person. This tool consists of 6 dimensions: bodily functions, mental wellbeing, meaningfulness, quality of life, participation, and daily functioning. Participants complete the questionnaire, resulting in a score between 0 and 10 for each dimension [30]. In our study, an adapted version will be used. Instead of completing a questionnaire consisting of 42 questions, the participants score each dimension from 0 to 10, as reported by van Velsen et al [31].

The SMAS-S is a questionnaire that measures 6 self-management abilities in older adults: taking initiative, investment behavior, variety, multifunctionality, self-efficacy, and positive frame of mind. It determines whether older adults need self-management courses [32].

#### Use of COUCH

The actual use will be determined using the platform’s log history. This outcome measure is defined as the frequency and duration of use overall, per week, and per session.

#### Demographics

Demographic data collected in the pretest questionnaire include gender, age, educational level, living situation, working status, attitude toward technology, self-reported level of physical activity, health literacy [33], and motivation level to live healthy. Attitude toward using technology and motivation level to live healthy will be explained in the following paragraphs.

To determine the participant’s attitude toward using technology, 4 items from Agarwal and Prasad [34] are included in the questionnaire. They developed and validated a new instrument consisting of 4 statements rated using a 7-point Likert scale, ranging from total disagreement to total agreement.

To get participants engaged in working on their health, it is important to determine their motivation to live healthy. With this information, the best suitable persuasive feature can be used in COUCH for each participant [31]. The motivation of an older adult to live healthy can be measured by a tool developed by van Velsen et al [31] based on the revised Sport Motivation Scale (SMS-II). The SMS-II was created and validated by Pelletier et al [35]. This questionnaire measures sport motivation using the Self-Determination Theory. The Self-Determination Theory distinguishes between 6 types of motivation: intrinsic motivation, extrinsic external regulation, extrinsic introjected regulation, extrinsic identified regulation, extrinsic integrated regulation, and a-motivation [36]. Those 6 types are included in the SMS-II tool. According to van Velsen et al [31], there are only 3 types of motivation in older adults to live healthy: intrinsic motivation, extrinsic external regulated, and a-motivation. They provided a set of 11 statements that will be used in our study. In our study, a fourth motivation type, dual motivation, will be included because some participants are not obviously intrinsically motivated nor externally motivated.

#### Applicability of the Virtual Coaches

The applicability of the virtual coaches will be measured using a rating scale and an adapted version of the Working Alliance Inventory Dutch version for use in the rehabilitation setting. This questionnaire will be completed for the 2 primary virtual coaches. This questionnaire measures how the patient feels about the therapeutic alliance: the better the therapeutic alliance, the more likely the patient will follow the treatment faithfully. Each participant will provide a score between 12 and 60: the higher the score, the more satisfied the participant is with the physical activity or nutrition coach and the more she/he trusts the coach [37].

### Sample Size

Because of the explorative character of this study, no sample size calculation was conducted beforehand. To answer the objectives of this study, the goal is to include 50 participants per country. So, in each round, 25 participants will be included per country. In our experience, participants are very enthusiastic to participate in this kind of evaluation with new technology before starting the study, but we expect that around 50% of the participants will drop out before the end of the implementation phase.

### Statistical Analysis

Statistical analyses will be performed using SPSS, version 19 for Windows (IBM Corp, Armonk, NY). For all analyses, the CIs will be set at 95%. Descriptive statistics, such as frequency, mean, SD, and percentages, will be used to describe demographics, user experience, actual use, and the applicability of the coaches.

The outcomes from the EQ-5D-5L, Positive Health questionnaire, and SMAS-S will be investigated using a mixed-model analysis for repeated measures to obtain the effect of using COUCH on the different measurements. The fixed factor will be the measurement time point (T0, T1, or T2). Post hoc comparisons will be conducted when required, and Sidak adjustments will be used to correct for multiple tests.

To assess the users’ interaction with the virtual coaches, the duration of the interaction (in seconds) and the number of dialogue steps with the coach will be used. With this analysis, we want to assess the effect of the conversation with the virtual coaches. To discover changes and possible trends, the duration of the interaction and number of dialogue steps will be analyzed for the two conditions. When the data follow a normal distribution, the outcome will be investigated using a paired *t* test; when the data are not normally distributed, a Wilcoxon signed-rank test will be performed.

### Ethics and Informed Consent

This study will be conducted according to the principles of the Declaration of Helsinki (64th WMA General Assembly, Fortaleza, Brazil, October 2013) and in accordance with the Medical Research Involving Human Subjects Act (Dutch law: Wet medisch-wetenschappelijk onderzoek met mensen). According to this law, this study does not require formal medical ethical approval in the Netherlands. This has been checked by the CMO Arnhem-Nijmegen (file number: 2019-5555). Each participant will give his/her informed consent on paper. See Multimedia Appendix 2 for the informed consent form.

## Results

Recruitment of participants will take place twice. The first round of recruitment occurred from December 1, 2019 to January 30, 2020 in the Netherlands, during which time we recruited 26 participants. The first round of recruitment is still ongoing in Scotland. The second round of recruitment will occur from March 1, 2020 to April 30, 2020. For each round, we will recruit 25 participants per country. Participants are recruited through a mass mailing to older adults, snowball sampling, and advertisements in local newspapers and social media. Participants contact the principal investigator to sign up for participation. The principal investigator sends interested individuals an information letter via email and checks the inclusion and exclusion criteria. If a participant is eligible and still wants to be enrolled in the study, the first visit is planned by the principal investigator, and the study starts.

**Figure 4:**
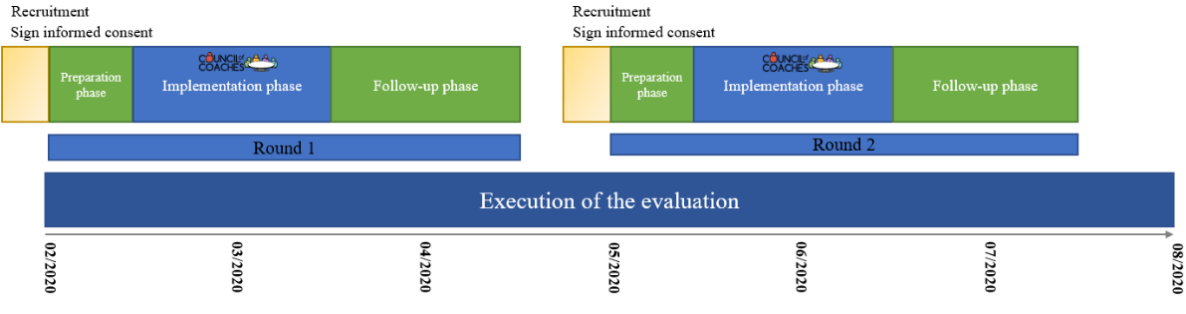
Timeline of the study evaluation period.

The first round of evaluation started on January 31, 2020. This round will last until April 15, 2020. The second round of evaluation will start in May 2020 and will last until July 2020. Figure 4 shows the planning of the evaluation. In August 2020, we plan to have the first results of this study.

## Discussion

### Overview

This protocol describes the final evaluation of the COUCH system. This study has the following strengths. First, the COUCH system was developed in collaboration with end users. Our expectation is that this will lead to fewer usability issues and better insight into the study’s primary outcome measures. McCurdie et al [38] reported that users identify key requirements that otherwise would entirely be neglected. Second, this evaluation will take place in the participants’ residence, a real-world setting, over a long period (5-9 weeks). This will provide a lot of information about how long the target group is willing to interact with a virtual coaching system and whether a virtual coaching system can lead to behavior change. Finally, the intervention will be personalized to the participants. We will start the evaluation with a 1-week baseline measurement, in which we will measure the participants’ activity level and eating patterns. With this information, we can personalize the physical activity and nutrition coaches for each participant, which will improve the effectiveness of COUCH. Lentferink et al [39] showed in their scoping review that personalized content improves adherence to eHealth technologies, which subsequently will lead to a more effective eHealth service.

### Limitations

However, this study also has some limitations. First, there will likely be selection bias. Participants contact the researchers to enroll in the study. We expect that these participants are already more motivated to live healthy or already live more healthily than the average older adult population and the average adult population with type 2 diabetes or chronic pain. Second, the content that will be ready at the start of the evaluation only lasts for 4 weeks. During the follow-up phase, no new content will be provided to the participants. This can influence the interaction frequency during the follow-up phase. Finally, this study will possibly have to deal with confounders, for example if users receive advice from their health care professionals or others about a healthy lifestyle. This occurs in real life. To handle this as best as possible, confounders such as these will be discussed with the users during the exit interview.

### Conclusions

This study will provide insight spanning many areas to improve the COUCH system, and it will contribute to further development of the system and to a better understanding of the value of virtual coaches for behavior change. In addition, the summative approach of this study protocol to evaluate an eHealth application in a real-world setting can be used to guide other eHealth evaluations.

## Appendix

Multimedia Appendix 1 Survey questions.

Multimedia Appendix 2 Informed consent form.

Multimedia Appendix 3 CONSORT-eHEALTH checklist (V 1.6.1).

## Abbreviations

COUCH: Council of Coaches.
eHealth: electronic health.
SMAS-S: Self-Management Ability Scale – short version.
SMS-II: revised Sports Motivation Scale.
SUS: System Usability Scale.
