# Supplementary material for: User Experience and Potential Health Effects of a Conversational Agent-Based Electronic Health Intervention: Protocol for an Observational Cohort Study
Source: JMIR Res Protoc. 2020 Apr 3;9(4):e16641. doi: 10.2196/16641 (PMC7165305; doi:10.2196/16641)
Supplement: Multimedia Appendix 1 [file resprot_v9i4e16641_app1.pdf]

Appendix 1: List of survey questions

T0 questionnaire

Demographics

1. What is your gender?
   - ☐ Male
   - ☐ Female

2. What is your age?

   ……………… years old

3. What is your highest level of education?
   - ☐ Primary school
   - ☐ Preparatory secondary vocational education
   - ☐ Higher general secondary education, pre-university education
   - ☐ Higher vocational education, university

4. What is your living situation?
   - ☐ Alone
   - ☐ Married/living together
   - ☐ Living together with my caregiver
   - ☐ Other

5. Which of the following categories best describes your work status?
   - ☐ Employed
   - ☐ Volunteer/caregiver
   - ☐ Retired
   - ☐ Job seeker
   - ☐ Other

Health literacy [1]

6. How often do you have problems learning about your medical condition because of difficulty understanding written information?

Never	Occasionally	Sometimes	Often	Always

7. How confident are you filling out medical forms by yourself?

Not at all	A little bit	Somewhat	Quite a bit	Extremely

8. How often do you have someone help you read hospital materials?

Never	Occasionally	Sometimes	Often	Always

Self-reported level of physical activity

9. How often do you engage in physical or sports activities?
   - ☐ Not at all
   - ☐ Not at all, but thinking about beginning
   - ☐ Less than 2.5 hours a week
   - ☐ More than 2.5 hours a week in the last six months
   - ☐ More than 2.5 hours a week for more than six months

Attitude toward using technology [2]

10. *How do you think about new technologies in general? Circle the answer that best fits your thoughts.*

    a. If I heard about a new information technology, I would look for ways to experiment with it.

Strongly disagree	Disagree	Slightly disagree	Neutral	Slightly agree	Agree	Strongly agree

    b. Among my peers, I am usually the first to try out new information technologies.

Strongly disagree	Disagree	Slightly disagree	Neutral	Slightly agree	Agree	Strongly agree

    c. In general, I am hesitant to try out new information technologies.

Strongly disagree	Disagree	Slightly disagree	Neutral	Slightly agree	Agree	Strongly agree

    d. I like to experiment with new information technologies.

Strongly disagree	Disagree	Slightly disagree	Neutral	Slightly agree	Agree	Strongly agree

Motivation to live healthy [3]

11. *We would like to look at your motivation to live a healthy life. We give you a number of statements, circle the answer that best fits your motivation.*

    a. I live healthy, because people around me reward me when I do.

Strongly disagree	Disagree	Slightly disagree	Neutral	Slightly agree	Agree	Strongly agree

    b. I live healthy, because I like to learn more about healthy living.

Strongly disagree	Disagree	Slightly disagree	Neutral	Slightly agree	Agree	Strongly agree

c. I live healthy, because I think others would disapprove of me if I did not.

Strongly disagree	Disagree	Slightly disagree	Neutral	Slightly agree	Agree	Strongly agree

d. I live healthy, because I like to discover new ways to lead a healthier life.

Strongly disagree	Disagree	Slightly disagree	Neutral	Slightly agree	Agree	Strongly agree

e. I live healthy, so that I get compliments from others.

Strongly disagree	Disagree	Slightly disagree	Neutral	Slightly agree	Agree	Strongly agree

f. I do not think a healthy life really fits me.

Strongly disagree	Disagree	Slightly disagree	Neutral	Slightly agree	Agree	Strongly agree

g. I live healthy, because I think it is one of the best ways to develop other sides of myself.

Strongly disagree	Disagree	Slightly disagree	Neutral	Slightly agree	Agree	Strongly agree

h. I live healthy, because I think it is very interesting to learn how to live a healthier live.

Strongly disagree	Disagree	Slightly disagree	Neutral	Slightly agree	Agree	Strongly agree

i. I live healthy, because the people that are important to me would be angry at me if I did not.

Strongly disagree	Disagree	Slightly disagree	Neutral	Slightly agree	Agree	Strongly agree

j. I live healthy, because I think it is a good way to develop my strong suits.

Strongly disagree	Disagree	Slightly disagree	Neutral	Slightly agree	Agree	Strongly agree

k. In the past, I had good reasons to live healthy, but nowadays I am doubting whether I want to continue healthy living.

Strongly disagree	Disagree	Slightly disagree	Neutral	Slightly agree	Agree	Strongly agree

Potential health effect questionnaires

Self-Management Ability Scale – Short version [4]

12. *The following questions are about all different kind of things in you daily living. E.g. things you do, your hobbies, your contacts with others, etc. The questions relate on your situation in general. Consider for example how it was in the last three months.*

a. How often do you take the initiative to keep yourself busy?

Never	Almost never	Sometimes	Regularly	Often	Very often

b. How often do you take initiative to get in touch with people who are dear to you?

Never	Almost never	Sometimes	Regularly	Often	Very often

c. How often do you make an effort to have friendly contacts with other people?

Never	Almost never	Sometimes	Regularly	Often	Very often

d. Do you ensure you have enough interests on a regular basis (such as a hobby) to keep you active?

Never	Almost never	Sometimes	Regularly	Often	Very often

e. Do you devote some time and attention to those who are dear to you in order to maintain good contact?

Never	Almost never	Sometimes	Regularly	Often	Very often

f. Do you keep busy with the things you are good at so that you stay good at them?

Never	Almost never	Sometimes	Regularly	Often	Very often

g. How many hobbies or activities do you have on a regular basis?

Zero	One	Two	Three or four	Five or six	More than six

h. Do you have different occasions on which you have friendly contact with others?

Zero	One	Two	Three or four	Five or six	More than six

i. Are there certain things that you are good at?

Zero	One	Two	Three or four	Five or six	More than six

j. The activities I enjoy, I do together with others.

Never	Almost never	Sometimes	Regularly	Often	Very often

k. I sometimes help the people I care about.

Never	Almost never	Sometimes	Regularly	Often	Very often

l. Others benefit from the things I do for my pleasure.

Never	Almost never	Sometimes	Regularly	Often	Very often

m. Are you able to find agreeable activities?

Never	Almost never	Sometimes	Regularly	Often	Very often

n. Are you able to have friendly contacts with others?

Never	Almost never	Sometimes	Regularly	Often	Very often

o. Are you able to let others know that you care about them?

Never	Almost never	Sometimes	Regularly	Often	Very often

p. When things go against you, how often do you think that it could always be worse?

Never	Almost never	Sometimes	Regularly	Often	Very often

q. When you have a bad day, how often do you think that things will be better tomorrow?

Never	Almost never	Sometimes	Regularly	Often	Very often

r. When things are not going so well, how often do you succeed in thinking positively?

Never	Almost never	Sometimes	Regularly	Often	Very often

Quality of life (EQ-5D-5L) [5]

13. *The following statements are about your quality of life. Under each heading, please tick the one box that best describes your health today.*

MOBILITY
- ☐ I have no problems in walking about
- ☐ I have slight problems in walking about
- ☐ I have moderate problems in walking about
- ☐ I have severe problems in walking about
- ☐ I am unable to walk about

SELF-CARE
- ☐ I have no problems washing or dressing myself
- ☐ I have slight problems washing or dressing myself
- ☐ I have moderate problems washing or dressing myself
- ☐ I have severe problems washing or dressing myself
- ☐ I am unable to wash or dress myself

USUAL ACTIVITIES (e.g. work, study, housework, family or leisure activities)
- ☐ I have no problems doing my usual activities
- ☐ I have slight problems doing my usual activities
- ☐ I have moderate problems doing my usual activities
- ☐ I have severe problems doing my usual activities
- ☐ I am unable to do my usual activities

PAIN/DISCOMFORT
- ☐ I have no pain or discomfort
- ☐ I have slight pain or discomfort
- ☐ I have moderate pain or discomfort
- ☐ I have severe pain or discomfort
- ☐ I have extreme pain or discomfort

ANXIETY/DEPRESSION
- ☐ I am not anxious or depressed
- ☐ I am slightly anxious or depressed
- ☐ I am moderately anxious or depressed
- ☐ I am severely anxious or depressed
- ☐ I am extremely anxious or depressed

14. We would like to know how good or bad your health is **today**. The scale on the right side of the page is numbered from 0 to 100. 100 means the **best** health you can imagine. 0 means the **worst** health you can imagine. Mark an X on the scale to indicate how your health is **today**, and please write the number you marked on the scale in the box below.

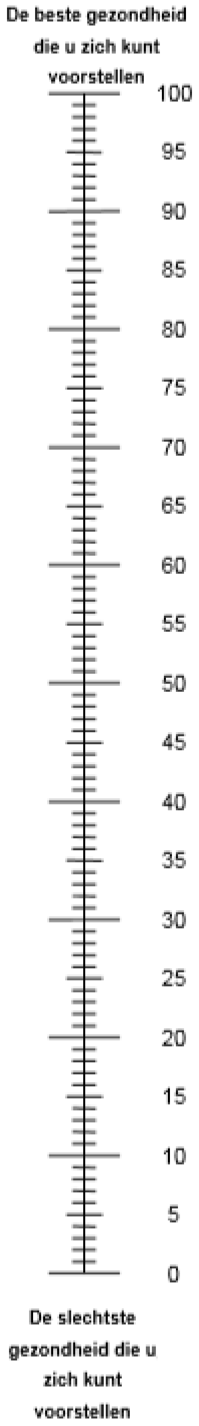

Positive Health dimensions [3,6]

15. *A number of questions about positive health will follow. For each question, circle the number that best fits your health. On a scale of 1 (low) to 10 (high).*

    a. How healthy do you think your body is? Do you feel fit? Are you in pain somewhere? Can you sleep and eat well?

1	2	3	4	5	6	7	8	9	10

    b. What do you think of your mental fitness? Can you concentrate well? Are you feeling well? Do you feel that you are in control of your life?

1	2	3	4	5	6	7	8	9	10

    c. We want to know how much satisfaction you get out of your life. Do you have a great zest for life? Do you have ideals that you want to achieve? Are you grateful for the things that life has given you?

1	2	3	4	5	6	7	8	9	10

    d. Can you indicate what the quality of your life is? Do you enjoy life? Do you feel safe? Do you have the idea that your life is in balance? Do you have a comfortable life?

1	2	3	4	5	6	7	8	9	10

    e. What do you think of your social life? Do you have enough friends? Do you have others to do fun things with? Do you get help if you need it? Do you feel life you belong somewhere?

1	2	3	4	5	6	7	8	9	10

    f. Can you indicate how well you can take care of yourself? Can you handle money well? Can you work? Do you know your limits? Can you ask for help if necessary?

1	2	3	4	5	6	7	8	9	10

16. Please score each coach on a scale of 1 (low) to 10 (high).

Olivia Simons (physical activity)

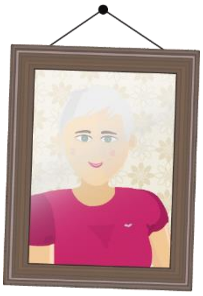

1 2 3 4 5 6 7 8 9 10

François Dubois (nutrition)

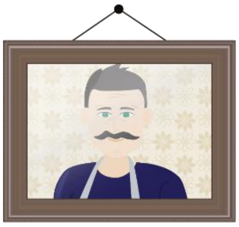

1 2 3 4 5 6 7 8 9 10

Emma Li (social)

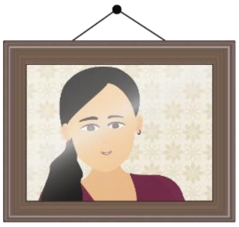

1 2 3 4 5 6 7 8 9 10

Helen Jones (cognitive)

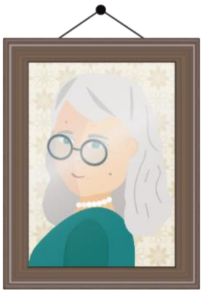

1 2 3 4 5 6 7 8 9 10

Carlos Silva (peer & support)

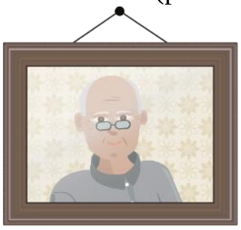

1 2 3 4 5 6 7 8 9 10

Rasmus Johansen (chronic pain)

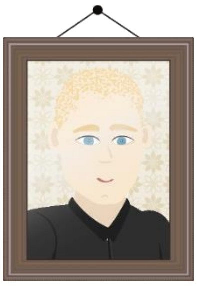

1 2 3 4 5 6 7 8 9 10

Katarzyna Kowalska (diabetes)

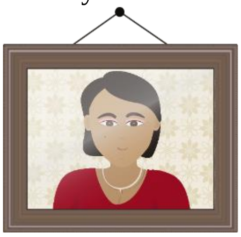

1 2 3 4 5 6 7 8 9 10

T1 Questionnaire

Potential health effect questionnaires

17. **Self-Management Ability Scale – Short version** [4] – see page 4 & 5
18. **Quality of life (EQ-5D-5L)** [5] – see page 6
19. **Positive Health dimensions** [3,6] – see page 7

User experience

Technology Acceptance Model [7–15]

20. *The following statements are about your user experience with COUCH. Circle the answer that best fits your experience.*

    a. The COUCH app was …

Disgusting						Enjoyable
1	2	3	4	5	6	7

    b. The COUCH app was …

Dull						Exciting
1	2	3	4	5	6	7

    c. The COUCH app was …

Unpleasant						Pleasant
1	2	3	4	5	6	7

    d. The COUCH app was …

Boring						Interesting
1	2	3	4	5	6	7

    e. The COUCH app looks clean.

Strongly disagree	Disagree	Slightly disagree	Neutral	Slightly agree	Agree	Strongly agree

    f. The COUCH app looks clear.

Strongly disagree	Disagree	Slightly disagree	Neutral	Slightly agree	Agree	Strongly agree

    g. The COUCH looks pleasant.

Strongly disagree	Disagree	Slightly disagree	Neutral	Slightly agree	Agree	Strongly agree

h. The COUCH app looks well balanced.

Strongly disagree	Disagree	Slightly disagree	Neutral	Slightly agree	Agree	Strongly agree

i. The COUCH app looks pretty.

Strongly disagree	Disagree	Slightly disagree	Neutral	Slightly agree	Agree	Strongly agree

j. I have a lot control over what I can do on the COUCH app.

Strongly disagree	Disagree	Slightly disagree	Neutral	Slightly agree	Agree	Strongly agree

k. On the COUCH app I can choose freely what I want to see.

Strongly disagree	Disagree	Slightly disagree	Neutral	Slightly agree	Agree	Strongly agree

l. I can determine for myself what happens on the COUCH app.

Strongly disagree	Disagree	Slightly disagree	Neutral	Slightly agree	Agree	Strongly agree

m. The security of the COUCH app gives me a comfortable feeling.

Strongly disagree	Disagree	Slightly disagree	Neutral	Slightly agree	Agree	Strongly agree

n. The law and security technology protect me well against problems with the COUCH app.

Strongly disagree	Disagree	Slightly disagree	Neutral	Slightly agree	Agree	Strongly agree

o. My personal data are well protected when I use the COUCH app.

Strongly disagree	Disagree	Slightly disagree	Neutral	Slightly agree	Agree	Strongly agree

p. The COUCH app is safe.

Strongly disagree	Disagree	Slightly disagree	Neutral	Slightly agree	Agree	Strongly agree

q. Using COUCH helps me understand my physical condition.

Strongly disagree	Disagree	Slightly disagree	Neutral	Slightly agree	Agree	Strongly agree

r. Using COUCH improves my physical condition.

Strongly disagree	Disagree	Slightly disagree	Neutral	Slightly agree	Agree	Strongly agree

s. Using COUCH improves my health.

Strongly disagree	Disagree	Slightly disagree	Neutral	Slightly agree	Agree	Strongly agree

t. Using COUCH gives me insight in my health.

Strongly disagree	Disagree	Slightly disagree	Neutral	Slightly agree	Agree	Strongly agree

u. It is clear and understandable how I can work with the COUCH app.

Strongly disagree	Disagree	Slightly disagree	Neutral	Slightly agree	Agree	Strongly agree

v. I do not have to think hard when working with the COUCH app.

Strongly disagree	Disagree	Slightly disagree	Neutral	Slightly agree	Agree	Strongly agree

w. I find the COUCH app easy to use.

Strongly disagree	Disagree	Slightly disagree	Neutral	Slightly agree	Agree	Strongly agree

x. I find it easy to get the COUCH app to do what I want it to do.

Strongly disagree	Disagree	Slightly disagree	Neutral	Slightly agree	Agree	Strongly agree

y. If the COUCH app would be available for me, I would definitely use it.

Strongly disagree	Disagree	Slightly disagree	Neutral	Slightly agree	Agree	Strongly agree

z. I would recommend the COUCH app to others.

Strongly disagree	Disagree	Slightly disagree	Neutral	Slightly agree	Agree	Strongly agree

aa. I hope that the COUCH app becomes available for me.

Strongly disagree	Disagree	Slightly disagree	Neutral	Slightly agree	Agree	Strongly agree

System Usability Scale [16]

21. *The following statements are about the user friendliness of the COUCH app. Indicate for each of the statements to what extent you agree with it.*

    a. I think that I would like to use the COUCH app frequently.

Strongly disagree	Disagree	Neutral	Agree	Strongly agree

    b. I found the COUCH app unnecessarily complex.

Strongly disagree	Disagree	Neutral	Agree	Strongly agree

    c. I thought the COUCH app was easy to use.

Strongly disagree	Disagree	Neutral	Agree	Strongly agree

    d. I think that I would need the support of a technical person to be able to use the COUCH app.

Strongly disagree	Disagree	Neutral	Agree	Strongly agree

    e. I found the various functions in the COUCH app were well integrated.

Strongly disagree	Disagree	Neutral	Agree	Strongly agree

    f. I thought there was too much inconsistency in the COUCH app.

Strongly disagree	Disagree	Neutral	Agree	Strongly agree

    g. I would imagine that most people would learn to use the COUCH app very quickly.

Strongly disagree	Disagree	Neutral	Agree	Strongly agree

    h. I found the COUCH app very cumbersome to use.

Strongly disagree	Disagree	Neutral	Agree	Strongly agree

    i. I felt very confident using the COUCH app.

Strongly disagree	Disagree	Neutral	Agree	Strongly agree

    j. I needed to learn a lot of things before I could get going with this system.

Strongly disagree	Disagree	Neutral	Agree	Strongly agree

Willingness-to-pay

22. Are you willing to pay for using the COUCH app?
    - ☐ Yes
    - ☐ No

23. Imagine the COUCH app is available in the Play Store/App Store of your phone, how much euros are you willing to pay for it?
    - ☐ 0 euros per month
    - ☐ 5 euros per month
    - ☐ 10 euros per month
    - ☐ 20 euros per month

Applicability of the virtual coaches [17]

24. *The following statements provide a description of how you can think or feel about the relationship with the primary virtual coaches. Indicate for each of the statements to what extent you agree with it.*

	**Physical activity coach (Olivia)**	**Nutrition coach (François)**
A result from communicating with the coach is that it is more clear what to do to improve my situation.	☐ Always<br>☐ Very often<br>☐ Often<br>☐ Sometimes<br>☐ Never	☐ Always<br>☐ Very often<br>☐ Often<br>☐ Sometimes<br>☐ Never
What I am doing via the coach, gives me new ways of looking at my problem.	☐ Always<br>☐ Very often<br>☐ Often<br>☐ Sometimes<br>☐ Never	☐ Always<br>☐ Very often<br>☐ Often<br>☐ Sometimes<br>☐ Never
I believe that the coach likes me.	☐ Always<br>☐ Very often<br>☐ Often<br>☐ Sometimes<br>☐ Never	☐ Always<br>☐ Very often<br>☐ Often<br>☐ Sometimes<br>☐ Never
The coach and I work together in determining my goals.	☐ Always<br>☐ Very often<br>☐ Often<br>☐ Sometimes<br>☐ Never	☐ Always<br>☐ Very often<br>☐ Often<br>☐ Sometimes<br>☐ Never
The coach and I respect each other.	☐ Always<br>☐ Very often<br>☐ Often<br>☐ Sometimes<br>☐ Never	☐ Always<br>☐ Very often<br>☐ Often<br>☐ Sometimes<br>☐ Never

The coach and I are working towards mutually agreed upon goals.	☐ Always<br>☐ Very often<br>☐ Often<br>☐ Sometimes<br>☐ Never	☐ Always<br>☐ Very often<br>☐ Often<br>☐ Sometimes<br>☐ Never
I feel that the coach appreciates me.	☐ Always<br>☐ Very often<br>☐ Often<br>☐ Sometimes<br>☐ Never	☐ Always<br>☐ Very often<br>☐ Often<br>☐ Sometimes<br>☐ Never
The coach and I agree on what is important for me to work on.	☐ Always<br>☐ Very often<br>☐ Often<br>☐ Sometimes<br>☐ Never	☐ Always<br>☐ Very often<br>☐ Often<br>☐ Sometimes<br>☐ Never
I think the coach cares about me even when I do things that (s)he does not approve.	☐ Always<br>☐ Very often<br>☐ Often<br>☐ Sometimes<br>☐ Never	☐ Always<br>☐ Very often<br>☐ Often<br>☐ Sometimes<br>☐ Never
I think the thing I do via/with the coach will help me achieve the changes I want.	☐ Always<br>☐ Very often<br>☐ Often<br>☐ Sometimes<br>☐ Never	☐ Always<br>☐ Very often<br>☐ Often<br>☐ Sometimes<br>☐ Never
The physical activity/diet coach and I have established a good understanding of the kind of changes that would be good for me.	☐ Always<br>☐ Very often<br>☐ Often<br>☐ Sometimes<br>☐ Never	☐ Always<br>☐ Very often<br>☐ Often<br>☐ Sometimes<br>☐ Never
I believe the way the physical activity/diet coach and I are working with my problem is correct.	☐ Always<br>☐ Very often<br>☐ Often<br>☐ Sometimes<br>☐ Never	☐ Always<br>☐ Very often<br>☐ Often<br>☐ Sometimes<br>☐ Never

25. *Please indicate for each of the coaches whether you talked to him/her, whether you, if possible, would like to talk for a longer period of time with him/her, and which score on a scale from 1 (low) to 10 (high) want to give him/her.*

a. Did you talk to the physical activity coach (Olivia Simons)?

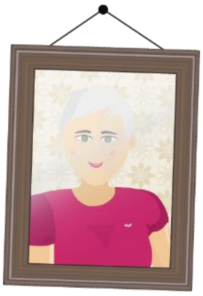

☐ Yes
☐ No

b. If possible, would you like to talk for a longer period of time with the physical activity coach (Olivia Simons)?

☐ Yes
☐ No

c. Please score the physical activity coach (Olivia Simons) on a scale from 1 (low) to 10 (high)

1 2 3 4 5 6 7 8 9 10

d. Did you talk to the nutrition coach (François Dubois)?

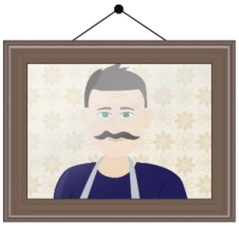

☐ Yes
☐ No

e. If possible, would you like to talk for a longer period of time with the nutrition coach (François Dubois)?

☐ Yes
☐ No

f. Please score the nutrition coach (François Dubois) on a scale from 1 (low) to 10 (high)

1 2 3 4 5 6 7 8 9 10

g. Did you talk to the social coach (Emma Li)?

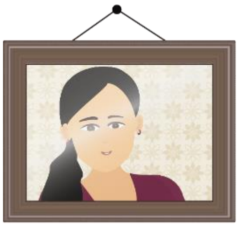

- ☐ Yes
- ☐ No

h. If possible, would you like to talk for a longer period of time with the social coach (Emma Li)?

- ☐ Yes
- ☐ No

i. Please score the social coach (Emma Li) on a scale from 1 (low) to 10 (high)

1 2 3 4 5 6 7 8 9 10

j. Did you talk to the cognitive coach (Helen Jones)?

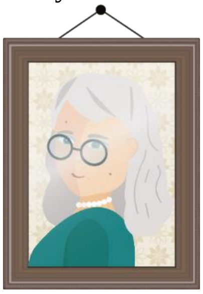

- ☐ Yes
- ☐ No

k. If possible, would you like to talk for a longer period of time with the cognitive coach (Helen Jones)?

- ☐ Yes
- ☐ No

l. Please score the cognitive coach (Helen Jones) on a scale from 1 (low) to 10 (high)

1 2 3 4 5 6 7 8 9 10

m. Did you talk to the peer & support coach (Carlos Silva)?

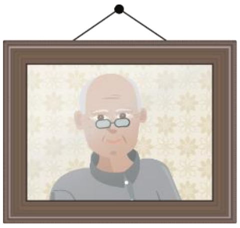

- ☐ Yes
- ☐ No

n. If possible, would you like to talk for a longer period of time with the peer & support coach (Carlos Silva)?
- ☐ Yes
- ☐ No

o. Please score the peer & support coach (Carlos Silva) on a scale from 1 (low) to 10 (high)

1 2 3 4 5 6 7 8 9 10

p. Did you talk to the chronic pain coach (Rasmus Johansen)?

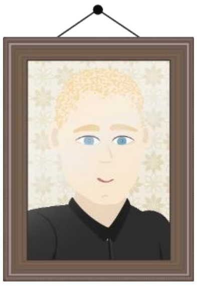

- ☐ Yes
- ☐ No

q. If possible, would you like to talk for a longer period of time with the chronic pain coach (Rasmus Johansen)?
- ☐ Yes
- ☐ No

r. Please score the chronic pain coach (Rasmus Johansen) on a scale from 1 (low) to 10 (high)

1 2 3 4 5 6 7 8 9 10

s. Did you talk to the diabetes coach (Katarzyna Kowalska)?

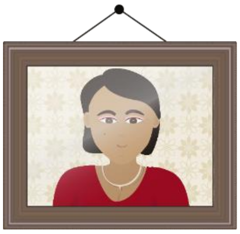

- ☐ Yes
- ☐ No

t. If possible, would you like to talk for a longer period of time with the diabetes coach (Katarzyna Kowalska)?

- ☐ Yes
- ☐ No

u. Please score the diabetes coach (Katarzyna Kowalska) on a scale from 1 (low) to 10 (high)

1 2 3 4 5 6 7 8 9 10

T2 Questionnaire

1. Did you use COUCH in the past four weeks?

   ☐ Yes

   ☐ No

2. Can you indicate why you have (not) used COUCH in the past four weeks?

____

____

____

____

____

Potential health effect questionnaires

3. **Self-Management Ability Scale – Short version** [4] – see page 4 & 5
4. **Quality of life (EQ-5D-5L)** [5] – see page 6
5. **Positive Health dimensions** [3,6] – see page 7

References

1. Chew LD, Bradley KA, Boyko EJ. Brief questions to identify patients with inadequate health literacy. Fam Med 2004;36(8):588–94. PMID:15343421
2. Agarwal R, Prasad J. A Conceptual and Operational Definition of Personal Innovativeness in the Domain of Information Technology. Inf Syst Res 1998 ;9(2):204–15. DOI:10.1287/isre.9.2.204
3. van Velsen L, Broekhuis M, Jansen-Kosterink S, op den Akker H. Tailoring Persuasive Electronic Health Strategies for Older Adults on the Basis of Personal Motivation: Web-Based Survey Study. J Med Internet Res 2019;21(9):e11759. DOI:10.2196/11759
4. Schuurmans H, Steverink N, Frieswijk N, Buunk BP, Slaets JPJ, Lindenberg S. How to Measure Self-management Abilities in Older People by Self-report. The Development of the SMAS-30. Qual Life Res 2005;14(10):2215–28. DOI:10.1007/s11136-005-8166-9
5. Van Reenen M, Janssen B. 2019. EQ-5D-5L User Guide: Basic information on how to use the EQ-5D-5L instrument. https://euroqol.org/wp-content/uploads/2019/09/EQ-5D-5L-English-User-Guide_version-3.0-Sept-2019-secured.pdf. Archived at: http://web.archive.org/web/20191002111745/https://euroqol.org/wp-content/uploads/2019/09/EQ-5D-5L-English-User-Guide_version-3.0-Sept-2019-secured.pdf
6. Huber M, van Vliet M, Giezenberg M, Winkens B, Heerkens Y, Dagnelie PC, et al. Towards a "patient-centred" operationalisation of the new dynamic concept of health: a mixed methods study. BMJ Open 2016;6(1):e010091. DOI: 10.1136/bmjopen-2015-010091
7. Davis FD. Perceived Usefulness, Perceived Ease of Use, and User Acceptance of Information Technology. MIS Q 1989;13(3):319. DOI:10.2307/249008
8. Davis FD, Bagozzi RP, Warshaw PR. User Acceptance of Computer Technology: A Comparison of Two Theoretical Models. Manage Sci 1989;35(8):982–1003. DOI:10.1287/mnsc.35.8.982
9. Van der Heijden H. User Acceptance of Hedonic Information Systems. MIS Q 2004;28(4):695–704. DOI:10.2307/25148660
10. Lavie T, Tractinsky N. Assessing dimensions of perceived visual aesthetics of web sites. Int J Hum Comput Stud 2004;60(3):269–98. DOI:10.1016/j.ijhcs.2003.09.002
11. van Velsen L, van der Geest T, van de Wijngaert L, van den Berg S, Steehouder M. Personalization has a Price, Controllability is the Currency: Predictors for the Intention to use Personalized eGovernment Websites. J Organ Comput Electron Commer 2015;25(1):76–97. DOI: 10.1080/10919392.2015.990782
12. Liu Y. Developing a Scale to Measure the Inactivity of Websites. J Advert Res. 2003;43(2):207–16. DOI:10.2501/jar-43-2-207-216
13. Harrison McKnight D, Choudhury V, Kacmar C. The impact of initial consumer trust on intentions to transact with a web site: a trust building model. J Strateg Inf Syst 2002;11(3–4):297–323. DOI:10.1016/S0963-8687(02)00020-3
14. Venkatesh V, Davis FD. A Theoretical Extension of the Technology Acceptance Model: Four Longitudinal Field Studies. Manage Sci 2000;46(2):186–204. DOI:10.1287/mnsc.46.2.186.11926
15. Gefen, Karahanna, Straub. Trust and TAM in Online Shopping: An Integrated Model. MIS Q 2003;27(1):51. DOI:10.2307/30036519
16. Brooke J. SUS - A quick and dirty usability scale. In: Jordan PW, Thomas B, McClelland IL, Weerdmeester B, editors. Usability evaluation in industry. 1st ed. London: Taylor & Francis; 1996. p. 189–194. ISBN:0748404600
17. Paap D, Schrier E, Dijkstra PU. Development and validation of the Working Alliance Inventory Dutch version for use in rehabilitation setting. Physiother Theory Pract 2018. DOI:10.1080/09593985.2018.1471112
