# Supplementary material for: User Experience and Potential Health Effects of a Conversational Agent-Based Electronic Health Intervention: Protocol for an Observational Cohort Study
Source: JMIR Res Protoc. 2020 Apr 3;9(4):e16641. doi: 10.2196/16641 (PMC7165305; doi:10.2196/16641)
Supplement: Multimedia Appendix 2 [file resprot_v9i4e16641_app2.pdf]

# Appendix 2: Informed consent form

<u>User experience, use and potential health effect of Council of Coaches</u>

- I have read the subject information form. I was also able to ask questions. My questions have been answered to my satisfaction. I had enough time to decide whether to participate.
- I know that participation is voluntary. I know that I may decide at any time not to participate after all or to withdraw from the study. I do not need to give a reason for this.
- I give permission for the collection and use of my data to answer the research question in this study
- I know that some people may have access to all my data to verify the study. These people are listed in this information sheet. I consent to the inspection by them.
- I ◻ **do**

  ◻ **do not**

  consent to being contacted again after this study for a follow-up study.
- I want to participate in this study.

Name of study subject:

Signature: Datum : ___ / ___ / _______

-----------------------------------------------------------------------------------------------------------------------------------------------

I hereby declare that I have fully informed this study subject about this study.

If information comes to light during the course of the study that could affect the study subject's consent, I will inform him/her of this in a timely fashion.

Name of investigator (or his/her representative):

Signature: Datum: ___ / ___ / _________

-----------------------------------------------------------------------------------------------------------------------------------------------

*The study subject will receive the full information sheet, together with a signed copy of the consent form.*
